# Preparation and Characterization of Atomic Oxygen-Resistant, Optically Transparent and Dimensionally Stable Copolyimide Films from Fluorinated Monomers and POSS-Substituted Diamine

**DOI:** 10.3390/polym16192845

**Published:** 2024-10-09

**Authors:** Zhenzhong Wang, Xiaolei Wang, Shunqi Yuan, Xi Ren, Changxu Yang, Shujun Han, Yuexin Qi, Duanyi Li, Jingang Liu

**Affiliations:** 1 Engineering Research Center of Ministry of Education for Geological Carbon Storage and Low Carbon Utilization of Resources, School of Materials Science and Technology, China University of Geosciences, Beijing 100083, China; 3003230013@email.cugb.edu.cn (Z.W.); 2103220038@email.cugb.edu.cn (X.W.); renxi@email.cugb.edu.cn (X.R.); 2103220040@email.cugb.edu.cn (C.Y.); 2103230036@email.cugb.edu.cn (S.H.); 2003230014@email.cugb.edu.cn (Y.Q.); 2103240041@email.cugb.edu.cn (D.L.); 2 RAYITEK Hi-Tech Film Company, Co. Ltd., Shenzhen 518105, China; sq.yuan@rayitek.cn

**Keywords:** polyimide, atomic oxygen, polyhedral oligomeric silsesquioxane, fluorine, optical properties, thermal properties

## Abstract

Optically transparent polyimide (PI) films with good atomic oxygen (AO) resistance have been paid extensive attention as thermal controls, optical substrates for solar cells or other components for low Earth orbit (LEO) space applications. However, for common PI films, it is usually quite difficult to achieve both high optical transparency and AO resistance and maintain the intrinsic thermal stability of the PI films at the same time. In the current work, we aimed to achieve the target by using the copolymerization methodology using the fluorinated dianhydride 9,9-bis(trifluoromethyl)xanthene-2,3,6,7-tetracarboxylic dianhydride (6FCDA), the fluorinated diamine 2,2-bis [4-(4-aminophenoxy)phenyl]hexafluoropropane (BDAF) and the polyhedral oligomeric silsesquioxane (POSS)-containing diamine *N*-[(heptaisobutyl-POSS)propyl]-3,5-diaminobenzamide (DABA-POSS) as the starting materials. The fluoro-containing monomers were used to endow the PI films with good optical and thermal properties, while the silicon-containing monomer was used to improve the AO resistance of the afforded PI films. Thus, the 6FCDA-based PI copolymers, including 6FCPI-1, 6FCPI-2 and 6FCPI-3, were prepared using a two-step chemical imidization procedure, respectively. For comparison, the analogous PIs, including 6FPI-1, 6FPI-2 and 6FPI-3, were correspondingly developed according to the same procedure except that 6FCDA was replaced by 4,4′-(hexafluoroisopropylidene)diphthalic anhydride (6FDA). Two referenced PI homopolymers were prepared from BDAF and 6FDA (PI-ref1) and 6FCDA (PI-ref2), respectively. The experimental results indicated that a good balance among thermal stability, optical transparency, and AO resistance was achieved by the 6FCDA-PI films. For example, the 6FCDA-PI films exhibited good thermal stability with glass transition temperatures (T_g_) up to 297.3 °C, good optical transparency with an optical transmittance at a wavelength of 450 nm (T_450_) higher than 62% and good AO resistance with the erosion yield (*E_y_*) as low as 1.7 × 10^−25^ cm^3^/atom at an AO irradiation fluence of 5.0 × 10^20^ atoms/cm^2^. The developed 6FCDA-PI films might find various applications in aerospace as solar sails, thermal control blankets, optical components and other functional materials.

## 1. Introduction

Optically transparent and low-colored polyimide (PI) films have been extensively investigated as functional components for space applications [1,2,3]. A series of optically transparent PI films, known as LaRC^TM^-CP1 and LaRC^TM^-CP2, were developed and reported by the Langley Research Center, National Aeronautics and Space Administration (NASA), USA, in the 1990s [4,5,6]. The LaRC^TM^-CP1 and LaRC^TM^-CP2 PI films are derived from the fluoro-containing dianhydride of 4,4′-(hexafluoroisopropylidene)diphthalic anhydride (6FDA) as well as the fluoro-containing diamine of 2,2-bis[4-(4-aminophenoxy)phenyl]hexafluoropropane (BDAF) for the former and the meta-substituted diamine of 1,3-bis(3-aminophenoxy)benzene (133APB) for the latter, respectively [7]. Especially, the LaRC^TM^-CP1 film has been proven to be stable in geostationary orbit (GEO) spacecraft for more than 10 years [8]. The highly electronegative fluorinated molecular structures endowed the LaRC^TM^-CP1 film with good optical transparency, low color intensity, low solar absorptivity and good ultraviolet (UV) irradiation resistance due to the great potentiality of the groups to reduce the charge-transfer (CT) interactions in the polymer chains [9]. In addition, the LaRC^TM^-CP1 film also exhibited good resistance to the charged particles’ irradiation, good thermal resistance (T_g_: 264.6 °C) and good tensile properties. However, the LaRC^TM^-CP1(6FDA-BDAF) film showed poor resistance to atomic oxygen (AO) irradiation, which is one of the most important space environments in low Earth orbit (LEO). The LaRC^TM^-CP1 film showed an AO erosion yield (*E_y_*) of 2.88 × 10^−24^ cm^3^/atom at an AO irradiation fluence of 5.0 × 10^20^ atoms/cm^2^ [10]. This value was comparable to that of the standard Kapton^®^ PI film (trademark of DuPont, Wilmington, DE, USA, *E_y_*: 3.0 × 10^−24^ cm^3^/atom) derived from pyromellitic dianhydride (PMDA) and 4,4′-oxydianiline (ODA) [11]. Thus, the LaRC^TM^-CP1 film could hardly be used in LEO applications, although it exhibited good stability in GEO environments. In view of the poor AO resistance of the CP-1 film, various structural and composition modifications, such as the incorporation of phosphorus elements [12,13,14,15], linear siloxane linkages [16], hyperbranched siloxane units [17], zirconium elements [18] and so on, have been performed. All these modifications were based on the strategy that the above special elements (P, Si, Zr, etc.) could react with AO in situ to form the passivation layers on the surface of the PI films [19,20]. However, these modifications could not easily achieve the excellent comprehensive properties of the PI films.

In the 2000s, a special siloxane structure of polyhedral oligomeric silsesquioxane (POSS) with a cage-like composition of silicon and oxygen elements was used for the development of AO-resistant PI films by the researchers in the Air Force Research Laboratory (AFRL), USA [21]. The Kapton^®^ films with main-chain POSS units (MC-POSS-Kapton^®^) and side-chain POSS units (SC-POSS-Kapton^®^) were successfully developed and evaluated either in the ground-simulated facility or in real LEO environments [22,23,24,25,26]. The POSS-containing PI films exhibited excellent AO resistance and good thermal and mechanical properties, especially for the SC-POSS-Kapton^®^ film [27]. However, the optical transparency of these two POSS-modified PI films was not addressed. Recently, Wright et al. reported on the chemical modification of fluorinated PI films via the incorporation of POSS side chains, and good optical transparency was obtained for the derived PI films [28]. Then, a POSS-modified PI film with excellent combined optical, thermal and AO resistance was developed and commercialized with the trademarks CORIN^®^ XLS (colorless organic/inorganic nanocomposite) by Mantech International Corp., Herndon, VA, USA, and POSS^®^ ImiClear by Hybrid Plastics, Hattiesburg, MS, USA, at the end of the 2000s [29,30]. In 2021, Wright and coworkers reported on the CORIN^®^ chemistry, which was prepared using a two-stage procedure [31]. First, a precursor was prepared using a chemical imidization procedure with 6FDA, HFBAPP (abbreviated as BDAF in this work), 3,5-diaminobenzoic acid (DBA) and the phthalic anhydride terminator as the starting materials. Then, the copolymer was reacted with aminopropylisobutyl POSS using dicyclohexyl carbodiimide (DCC) as the acylation reagent and dichloromethane (DCM) and N,N-dimethylacetamide (DMAc) as the solvents. The CORIN^®^ resin was then obtained as pale-yellow powder from which the clear and colorless CORIN^®^ films could be further obtained. The CORIN^®^ film showed a glass transition temperature (T_g_) of 266 °C, a linear coefficient of thermal expansion (CTE) of 68.0 × 10^−6^/K in the temperature range of 125–175 °C, and the average optical transmittance of 88% in the wavelength range of 400–780 nm. In addition, the CORIN^®^ films showed excellent AO resistance both in the ground simulation device and in the real LEO tests, and the AO erosion yield of the CORIN^®^ films was about two orders of magnitude lower than that of the referenced Kapton^®^ films under the same conditions.

In the current work, we attempted to prepare the CORIN^®^ resins using a modified version of the procedure reported in ref. [31], in which the target polymers were directly prepared via the polycondensation reaction of 6FDA, BDAF and the POSS-containing diamine of *N*-[(heptaisobutyl-POSS)propyl]-3,5-diaminobenzamide (DABA-POSS) via the chemical imidization procedure without controlling the molecular weights. The prepared CORIN^®^-like resins and the derived PI films (6FPI-1~6FPI-3) were used as the references. The main purpose was to develop a series of new fluorinated PI resins and films with CORIN^®^-like structures except that the linear 6FDA dianhydride was replaced with a multi-ring 9,9-bis(trifluoromethyl)xanthene-2,3,6,7-tetracarboxylic dianhydride (6FCDA) dianhydride. This molecular design might efficiently improve the high-temperature dimensional stability of the derived PI films while maintaining the intrinsic optical and thermal properties of the polymers. This modification might expand the applications of functional PI films for space environments and enhance the reliability of spacecraft. The effects of the designed molecular structures on the thermal, optical and AO resistance properties of the derived fluorinated PI films (6FCPI-1~6FCPI-3) were investigated in detail.

## 2. Materials and Methods

### 2.1. Materials

The commercially available 9,9-bis(trifluoromethyl)xanthene-2,3,6,7-tetracarboxylic dianhydride (6FCDA) and 4,4’-(hexafluoroisopropylidene)diphthalic anhydride (6FDA) monomers were purchased from ChinaTech Chem. Co. Ltd. (Tianjin, China) and dried in vacuo at 180 °C for 24 h before use. The white crystals of the POSS-containing diamine, N-[(heptaisobutyl-POSS)propyl]-3,5-diaminobenzamide (DABA-POSS), was synthesized in our laboratory according to the literature [25] and purified via recrystallization from ethanol and de-colored with active carbon powder. The fluoro-containing diamine of 2,2-bis[4-(4-aminophenoxy)phenyl]hexafluoropropane (BDAF or HFBAPP) was obtained from Changzhou Sunlight Pharmaceutical Co., Ltd. (Changzhou, China) and used directly. The highly dried organic solvents, including N,N-dimethylformamide (DMF; purity: 99.9%; water content < 50 ppm), N,N-dimethylacetamide (DMAc; purity: 99.9%; water content < 50 ppm) and N-methyl-2-pyrrolidone (NMP; purity: 99.9%; water content < 50 ppm) were purchased from Innochem Science & Technology Co. Ltd. (Beijing, China) and used as received. The other analytical reagents were all purchased from Innochem Science & Technology Co. Ltd. (Beijing, China) and were used as received.

### 2.2. Characterization Methods

PI resins: The number average (M_n_) and weight average (M_w_) molecular masses were measured using a gel permeation chromatography (GPC) system (Shimadzu, Kyoto, Japan) with the HPLC grade of NMP as the mobile phase and with a Shodex KF-804 column (Tokyo, Japan). Hydrogen nuclear magnetic resonance (^1^H-NMR) spectra were obtained using an AV 400 spectrometer (Ettlingen, Germany) at 400 MHz in deuterated dimethyl sulfoxide (DMSO-d_6_) for the 6FPI and deuterated N,N-dimethylformamide (DMF-d_7_) for the 6FCPI resins, respectively. Wide-angle X-ray diffraction (XRD) spectra were obtained using a Rigaku D/max-2500 X-ray diffractometer (Tokyo, Japan) at 40 kV and 200 mA with Cu-Kα1 radiation. The solubility was tested by mixing 1.0 g of the PI resins with 9.0 g of the solvents to afford a mixture at the solid content of 10 wt%, followed by stirring for 24 h at room temperature. The solubility was evaluated as three different grades of completely soluble (++), partially soluble (+) and insoluble (−).

PI films: Fourier-transform infrared (FTIR) spectra were obtained using an Iraffinity-1S FT-IR spectrometer (Shimadzu, Kyoto, Japan) from a wavenumber of 4000 cm^−1^ to 500 cm^−1^ with a resolution of 4 cm^−1^. Ultraviolet–visible (UV-Vis) spectra were obtained using a Hitachi U-3210 spectrophotometer (Tokyo, Japan) at room temperature with samples at a thickness of around 25 μm. Refractive indices, including the in-plane refractive indices (n_TE_) and out-of-plane refractive indices (n_TM_), were measured on a Metricon Model 2010/M prism coupler (Pennington, NJ, USA) at a wavelength of 632.8 nm. Using the n_TE_ and n_TM_ results, the average refractive indices (n_av_) and the birefringence (Δn) were calculated as n_av_ = [(2n_TE_^2^ + n_TM_^2^)/3]^1/2^ and Δn = n_TE-_n_TM_, respectively. The CIE (International Commission on Illumination) color parameters, including the lightness (L*), the red-green indices (a*) and the yellow-blue indices (b*) were determined using an X-rite color i7 spectrophotometer (Grand Rapids, MI, USA) with samples at a thickness of around 50 μm. Field-emission scanning electron microscope (FE-SEM) measurements were conducted using a Technex Lab Tiny-SEM 1540 apparatus (Tokyo, Japan) with an accelerating voltage of 15 KV. Gold metallization of the samples was carried out with a gold thickness of around 20 nm. Atomic force microscopy (AFM) measurements were conducted using a Bruker Multimode 8 AFM microscope (Santa Barbara, CA, USA) with tapping mode. The Scanasyst-Air cantilever model (Bruker, Santa Barbara, CA, USA) with a resonance frequency of 70 kHz, spring constant of 0.4 N/m and size of 115 μm × 25 μm × 650 nm) was used. X-ray photoelectron spectroscopy (XPS) measurements were conducted using a Thermo Fisher Scientific ESCALab220i-XL electron spectrometer (Waltham, MA, USA) with monochromatic MgKα radiation. Thermogravimetric analysis (TGA) and the derivative TGA (DTG) plots were achieved using a TA Q50 thermal analysis system (New Castle, DE, USA) from room temperature to 750 °C at a heating rate of 20 °C/min in nitrogen with a gas flow of 20 mL/min. The sample weights were around 10 mg. Differential scanning calorimetry (DSC) plots were obtained using a TA Q100 thermal analysis system (New Castle, DE, USA) from room temperature to 400 °C at a heating rate of 10 °C/min in nitrogen with a gas flow of 20 mL/min. Dynamic mechanical analysis (DMA) plots were obtained using a TA Q800 thermal analysis system (New Castle, DE, USA) from room temperature to 400 °C at a heating rate of 5 °C/min and a frequency of 1Hz in nitrogen. Thermo-mechanical analysis (TMA) plots were obtained using a TMA402F3 thermal analysis system (NETZSCH, Selb, Germany) from room temperature to 400 °C at a heating rate of 5 °C/min in a nitrogen atmosphere. The linear coefficients of linear thermal expansion (CTE) values were detected in the range of 125–175 °C. The dielectric properties were tested on an Agilent 4294A precise impedance analyzer (Palo Alto, CA, USA) at room temperature at the frequency 10 GHz. The samples were dried at 100 °C for 1 h to remove the absorbed moisture prior to testing. Before the measurement, silver electrodes were fabricated on both sides of the samples using conductive silver paint and then accurately cut into small sheets (1 cm × 1 cm × 3 mm). All measurements were performed in an air atmosphere and at room temperature. The dielectric constant (*D_k_*) and dielectric dissipation factor (*D_f_*) data of the PI films were the average data of five parallel samples. The AO erosion behaviors were evaluated in a ground-simulated facility in Beijing Institute of Spacecraft Environment Engineering [32] with PI samples of 20 (length) × 20 (width) × 0.05 (thickness) mm^3^ in size. The AO fluence was controlled to be 5.0 × 10^20^ atoms/cm^2^. The AO erosion yield of the PI samples, *E_y_*, was calculated using Equation (1) [33]:

(1) $$E_{y}=\frac{∆M_{s}}{A_{s}\rho_{s}F}$$

where *E_y_* stands for the erosion yields of the PIs (cm^3^/atom); Δ*M_s_* stands for the mass loss of the PI samples (g); *A_s_* stands for the surface area of the PI samples exposed to AO (cm^2^) (2 cm × 2 cm in the present work); *ρ_s_* stands for the density of the samples (g/cm^3^); and *F* stands for the AO fluence (atoms/cm^2^) in the test (5.0 × 10^20^ atoms/cm^2^ in the present work). Kapton^®^ film was used as the reference, which has a constant *E_y_* value of 3.0 × 10^−24^ cm^3^/atom [34]. All the PI samples in the test are supposed to possess a similar density and exposure area to the referenced Kapton^®^ films. Therefore, Equation (1) could be simplified as Equation (2):

(2) $$E_{y}=\frac{∆M_{s}}{∆M_{Kapton}}E_{Kapton}$$

where *E_Kapton_* stands for the *E_y_* value of the Kapton^®^ film, 3.0 × 10^−24^ cm^3^/atom; Δ*M_Kapton_* is the mass loss of the Kapton^®^ references.

### 2.3. PI Resin Synthesis and Film Preparation

Two series of PI resins (six samples in total), including three 6FDA-based PIs (6FPI-1~6FPI-3) and three 6FCDA-based PIs (6FCPI-1~6FCPI-3) were prepared, respectively, according to the recipes shown in Table 1.

6FCPI-3 was used to illustrate the synthesis pathway for the resins. First, the diamine mixture of BDAF (17.3681 g, 33.5 mmol) and DABA-POSS (16.6437 g, 16.5 mmol) was added into ultra-dry NMP (150.0 g) contained in a 1000 mL four-necked glass vessel equipped with a mechanical stirrer, a nitrogen inlet and outlet and a thermometer. A wine-red color diamine solution was obtained after stirring at room temperature for 30 min under a dry nitrogen atmosphere. 6FCDA dianhydride (22.9110 g, 50 mmol) was then added into the diamine solution together with additional NMP (20.8 g). A reaction system with a solid content of 25 wt% was then obtained, which was stirred at room temperature for another 24 h to afford a viscous poly(amic acid) (PAA) solution. Acetic anhydride (Ac_2_O; 30.6 g, 300 mmol) and pyridine (Py; 19.8 g, 250 mmol) were then added to the PAA system. The reaction mixture was stirred at room temperature for another 24 h to afford the chemically imidized PI solution. The PI solution was then slowly added into an aqueous ethanol solution (70 vol%) to afford the filament resin. The resin was further immersed into a pure ethanol solution for another 24 h to thoroughly extract the residual NMP. The resin was naturally dried to evaporate most of the ethanol and then dried at 120 °C in vacuo overnight. Finally, the pale-yellow colored 6FCPI-3 resin was obtained: yield: 53.86 g (97.7%); M_n_: 4.60 × 10^4^ g/mol; M_w_: 9.05 × 10^4^ g/mol; polydispersity index (PDI): 1.97.

The thoroughly dried 6FCPI-3 resin was dissolved in ultra-dry DMAc at a solid content of 20 wt% to afford a clear solution. The homogeneous solution was purified via filtration using a 1.0 μm Teflon syringe filter. The obtained 6FCPI-3 solution was coated on a clean borosilicate glass substrate (20 cm × 20 cm × 5 mm) with a thickness-adjustable doctor knife. The substrate was placed in a nitrogen-circulating oven and then thermally baked with the heating procedure of 80 °C/2 h, 120 °C/1 h, 180 °C/1 h and 250 °C/1 h. After the drying treatment, the glass substrate was immersed into deionized water. The free-standing 6FCPI-3 film was obtained by peeling it from the substrate. Then, the film was dried at 120 °C in vacuo.

The other copolymerized resins and films were prepared according to the similar procedure mentioned above using the recipes shown in Table 1.

Two referenced homopolymerized PI resins and films, including PI-ref1 and PI-ref2, were also prepared from BDAF and 6FDA for the former and 6FCDA for the latter, respectively, using the similar procedure mentioned above.

## 3. Results and Discussion

### 3.1. PI Resin Synthesis and Film Preparation

**Figure 1:**
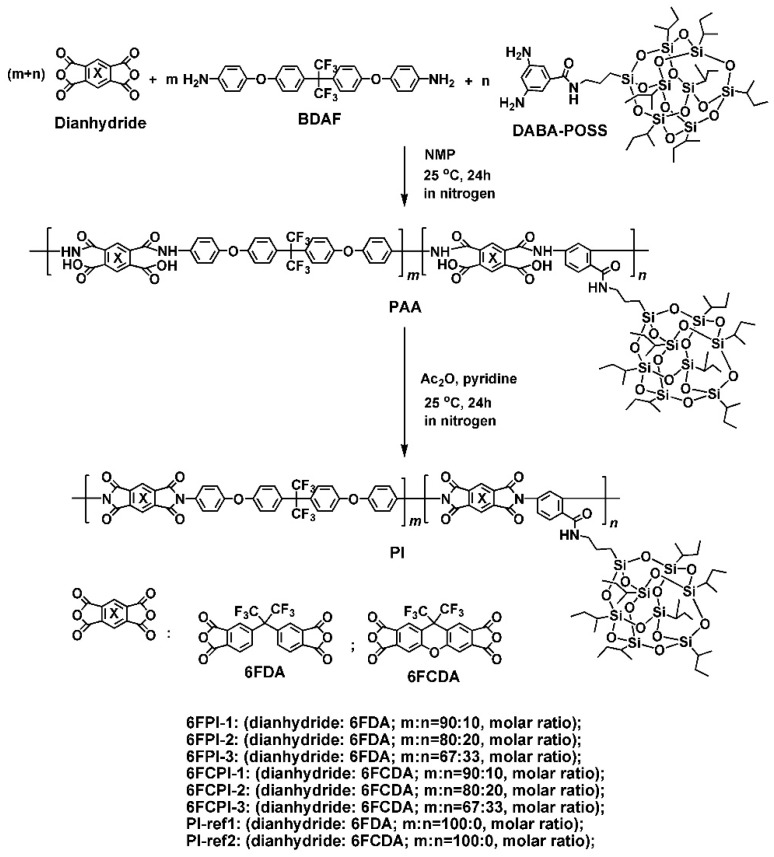
Preparation of PI and referenced PI resins.

Six copolymerized and two referenced homopolymerized PI resins were prepared according to the procedure shown in Figure 1.

**Figure 2:**
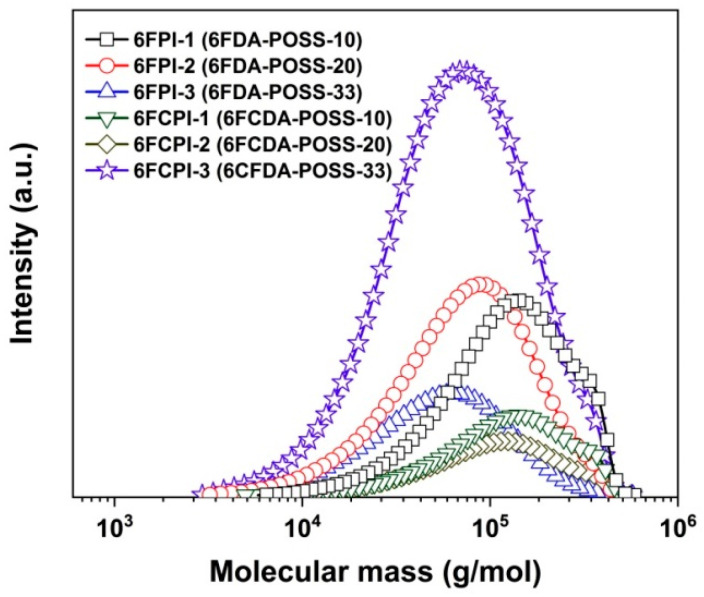
GPC plots of PI resins.

All the derived PI resins showed good solubility in the polymerization medium, and no gelation or precipitation occurred during the chemical imidization courses. This is mainly due to the existence of the bulky hexafluoroisopropylidene linkages or the lateral POSS side chains. The derived PI resins exhibited moderate to high M_n_ and M_w_ values and relatively narrow polydispersity indices (PDIs), as shown in Table 2. The following conclusions could be deduced from these molecular mass data: First, by comparing the molecular masses of the homopolymer and the copolymers, it could be seen that the molecular mass of the polymers decreased with the incorporation of the DABA-POSS diamine. Secondly, the molecular mass of the polymers decreased with the increasing molar proportion of DABA-POSS in the diamine components. For example, for the 6FCPI polymers, as the mole proportion of DABA-POSS in the diamine components increased from 0 to 33 mol%, the M_w_ values decreased from 15.30 × 10^4^ g/mol for PI-ref2 to 14.34 × 10^4^ g/mol (6FCPI-1), 12.59 × 10^4^ g/mol (6FCPI-2) and 9.05 × 10^4^ g/mol (6FCPI-3), respectively, as shown in the GPC plots of the PI resins (Figure 2). The same trend was observed for the 6FPI polymers. This might be due to the fact that the polymerization reactivity of the DABA-POSS diamine was less than that of BDAF. The electron-withdrawing amide bonds in the DABA-POSS structure reduced the densities of the electrons on the amino group, which reduced the reactivity of the amines when attacking the anhydride groups. On the other hand, the existence of bulky POSS side chains might reduce the reaction probabilities of the DABA-POSS diamine with the dianhydrides due to steric effects. Nevertheless, M_n_ values higher than 10^4^ g/mol could ensure that the prepared PI films possessed good toughness and flexibility, which could meet the property requirements of practical applications.

**Figure 3:**
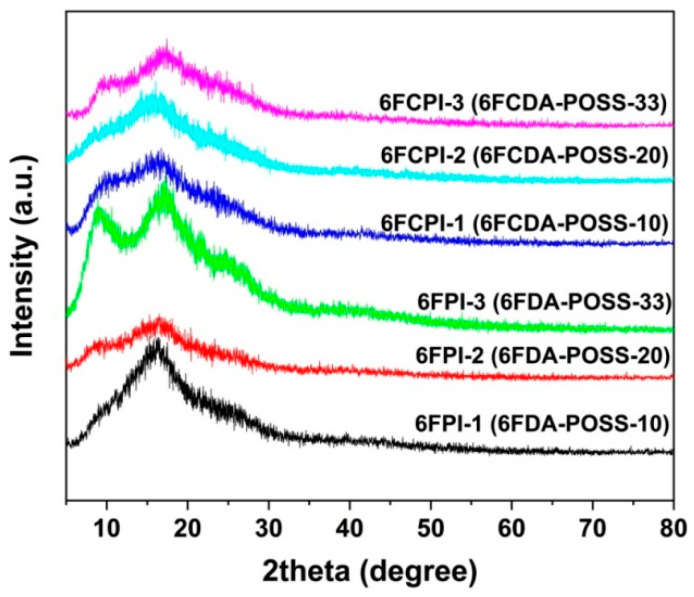
XRD spectra of PI resins.

The solubility of the PI resins was tested, and the results are tabulated in Table 2. All the PI resins exhibited good solubility in the polar aprotic solvents, such as NMP, DMAc and DMF. The 6FPI resins were also soluble in DMSO, while the analogous 6FCPI resins were only partially soluble in the solvent. The incorporation of the DABA-POSS units decreased the solubility of the PI resins. For example, the PI-ref1 was soluble in cyclopentanone, while the 6FPI-1 and 6FPI-2 resins were only partially soluble, and 6FPI-3 was not soluble in the solvent. The good solubility of the PI resins in the aprotic solvents was ascribed to the synergistic effects of the bulky hexafluoroisopropylidene units both in the dianhydride and diamine moieties, the flexible ether linkages (–O–) in the BDAF diamine moiety or the lateral POSS side chains in the DABA-POSS diamine moiety. The inferior solubility of the 6FCPI resins was due to the relatively higher packing density of the molecular chains in the polymers caused by the planar xanthene units in the 6FCDA moiety [34]. In addition, the rigid-rod amide linkages in the DABA-POSS diamine were conducive to the ordered stacking of the molecular chains, which might induce the local ordered or crystalline structures within the polymer molecular chains. This was verified by the XRD results of the PI resins, as shown in Figure 3. In the XRD pattern, it can be clearly observed that obvious crystalline peaks appear in the spectra of 6FPI-3 and 6FCPI-3 with the highest DABA-POSS contents. The existence of the locally crystalline regions was favorable for the obstruction of solvent penetration and, thus, reduced the solubility of the resin in the organic solvent. This was consistent with the solubility test results. In addition, it could be deduced that the existence of the crystalline region in the polymer chains might be unfavorable for the transmission of the visible lights, which might reduce the optical transmittance of the 6FPI-3 and 6FCPI-3 films. This will be discussed in detail in the subsequent optical performance analysis.

**Figure 4:**
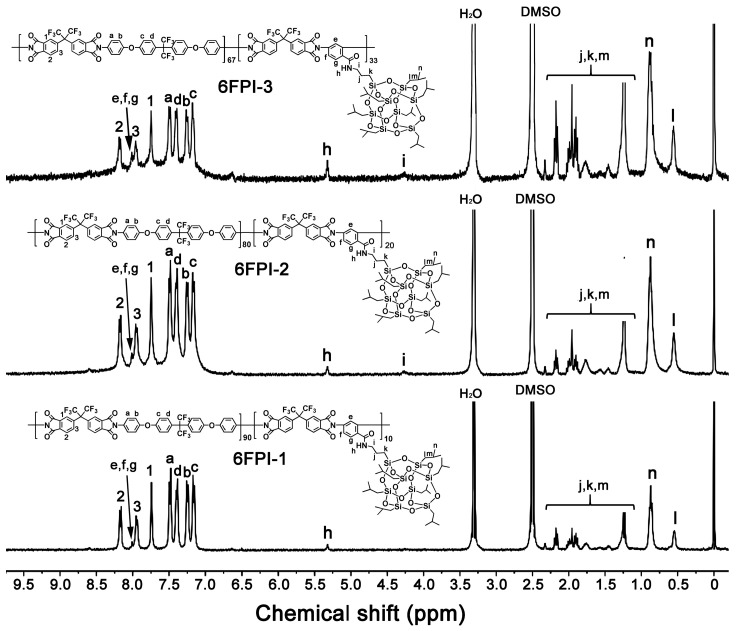
^1^H-NMR spectra of 6FPI resins in DMSO-d_6_.

**Figure 5:**
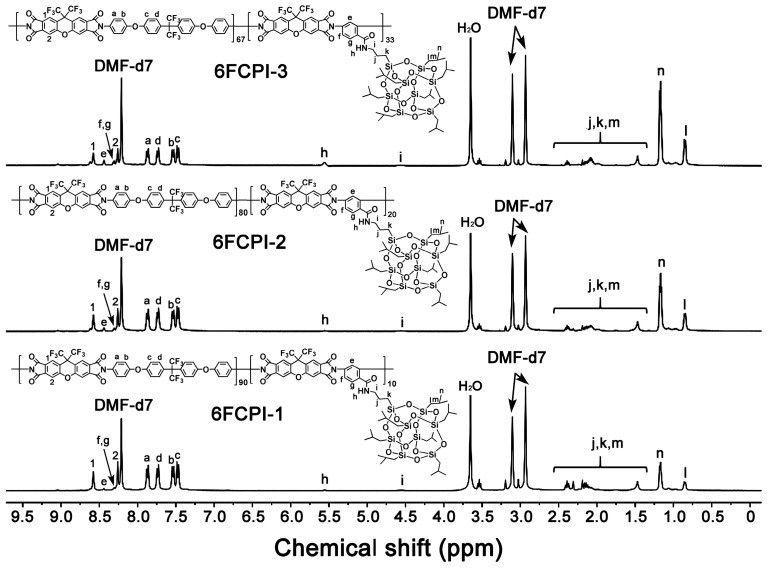
^1^H-NMR spectra of 6FCPI resins in DMF-d_7_.

Since the 6FCPI resins showed poor solubility in DMSO, the ^1^H-NMR spectra of the resins were measured with the DMF-d7 solvent, while the 6FPI resins were measured with the more common DMSO-d_6_. Figure 4 and Figure 5 show the ^1^H-NMR spectra of 6FPI and 6FCPI resins, respectively [10,34]. For both resin systems, the absorption of the protons could be clearly divided into three parts. The first part was located in the downfield areas in the chemical shift range of 7.0–8.5 ppm, the second one was in the range of 4.0–5.5 ppm, and the third one was in the upfield areas from 0.5 to 2.5 ppm. The protons ortho-substituted to the electron-withdrawing hexafluoroisopropylidene units (H_1_, H_2_ and H_3_ for 6FPI; H_1_ and H_2_ for 6FCPI) and in the amide-substituted phenyl (H_e~g_) revealed the absorptions at the furthest downfield fields in the spectra, while the protons in the isobutyl groups in the POSS units (H_j_~H_n_) showed the absorptions at the furthest upfield areas in the spectra. The protons in (H_h_) and close (H_i_) to the amide groups exhibited the absorptions in the middle area in the spectra. This information is in good agreement with the expected structures of the PIs, indicating the successful preparation of the target polymers.

**Figure 6:**
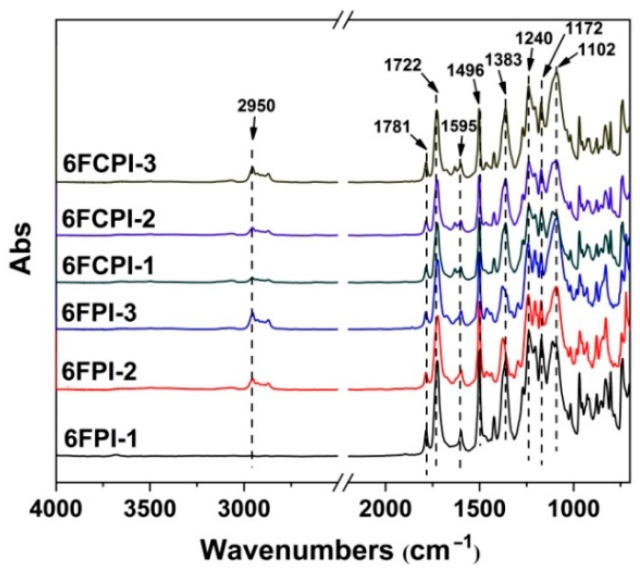
FTIR spectra of PI films.

The PI films were then fabricated by thermally casting the PI solution at elevated temperatures [10,34]. All the derived films exhibited good flexibility and toughness. The chemical structures of the films were confirmed by the FTIR measurements shown in Figure 6. The characteristic absorptions of imide rings, including the asymmetrical and symmetrical carbonyl stretching vibrations at 1780 cm^−1^ and 1722 cm^−1^, respectively, and the C–N stretching vibrations at 1383 cm^−1^, were all clearly detected. In addition, the characteristic absorption peaks of the other substituents—such as the ones at 1595 cm^−1^ and 1496 cm^−1^ ascribed to the stretching vibration of C=C in benzene rings, the ones at 2933 cm^−1^ due to the stretching vibration of saturated C-H bonds in the isobutyl groups of the POSS rings, the ones at 1102 cm^−1^ due to the stretching vibration of the Si-O linkages in the POSS units, the ones at 1172 cm^−1^ due to the stretching vibration of C–F bonds in the hexafluoroisopropylidene units and the ones at 1240 cm^−1^ due to the stretching vibration of C–O–C linkages—were also observed. The structural features further confirmed the successful preparation of the target polymers.

### 3.2. Thermal and Dielectric Properties

**Figure 7:**
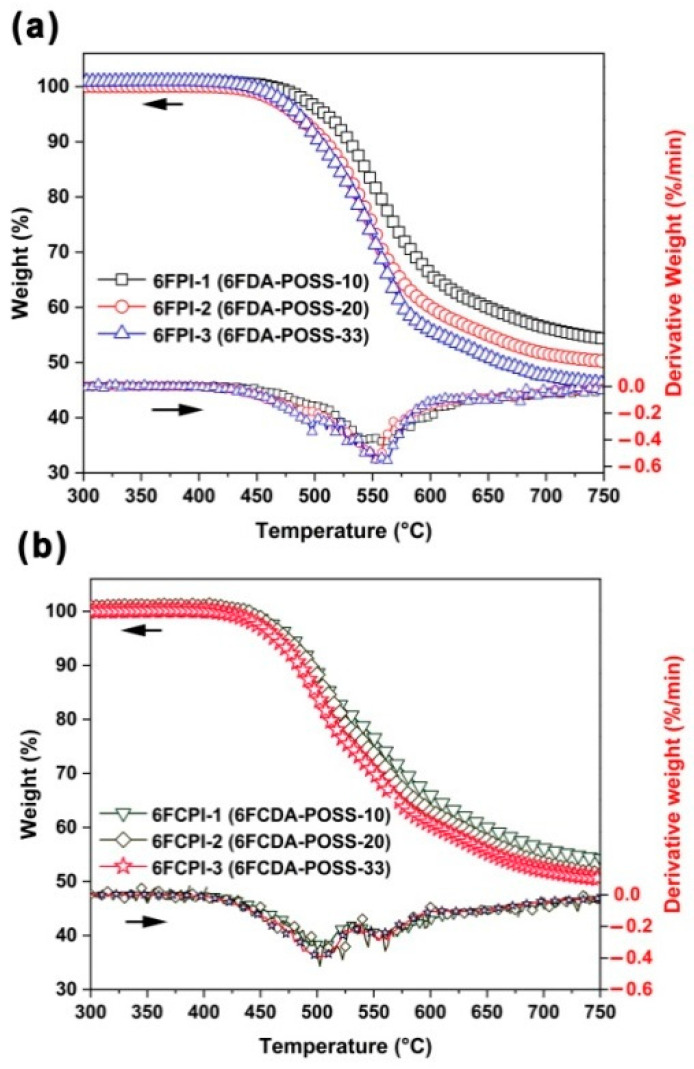
TGA and DTG curves of PI films in nitrogen. (**a**) 6FPI; (**b**) 6FCPI.

Figure 7 depicts the TGA and the derivative TG (DTG) plots of the 6FPI and 6FCPI films, and the thermal data are summarized in Table 3. By analyzing the spectra and the corresponding thermal data, the following conclusions could be deduced: Firstly, by comparing the 5% weight loss temperatures (T_5%_) of the copolymerized PIs with those of the homopolymerized reference PIs, it could be seen that the T_5%_ values of the PI films were significantly reduced after the incorporation of the POSS components. For example, the T_5%_ value of the PI-ref2 (6FCDA-BDAF) film was 516.8 °C, while the 6FCPI-1~6FCPI-3 films with the incorporation of 10–33 mol% of POSS components in the diamine units showed T_5%_ values of 477.4 °C, 472.5 °C and 463.3 °C, respectively. A similar trend was observed for the 6FPI systems. In addition, the current PI films with the covalently linked POSS in the structures showed lower T_5%_ values than the nanocomposite films reported in ref. [13]. For example, most of the nanocomposite films containing trisilanolphenyl-POSS (TSP–POSS) as the filler showed T_5%_ values higher than 500 °C, which were all higher than those of the current PI films. This is mainly because the TSP-POSS additives showed good thermal stability with a T_5%_ value higher than 500 °C, while the covalently linked DABA-POSS only showed a T_5%_ value around 350 °C [13].

Secondly, as expected, the thermal decomposition temperatures of the 6FCPI films based on 6FCDA dianhydride were lower than those of the 6FPI films based on 6FDA. This phenomenon has been widely reported in the literature [35], which was mainly attributed to the unstable feature of the xanthene rings at elevated temperatures. This could be seen in the DTG curves shown in Figure 7b, in which the obvious two-stage thermal decomposition behaviors were observed for the 6FCPI films. The temperatures corresponding to the first rapidest thermal decomposition rate (T_max1_) and the second decomposition fastest temperature (T_max2_) were in the range of 502–505 °C and 555–560 °C, respectively. In contrast, the 6FPI films mainly exhibited a single-stage thermal decomposition behavior with T_max1_ values of about 552–560 °C. The films showed residual weight ratios at 750 °C (R_w750_) of 46.1–54.3 wt% for 6FPIs and 50.6–54.2 wt% for 6FCPIs, respectively.

**Figure 8:**
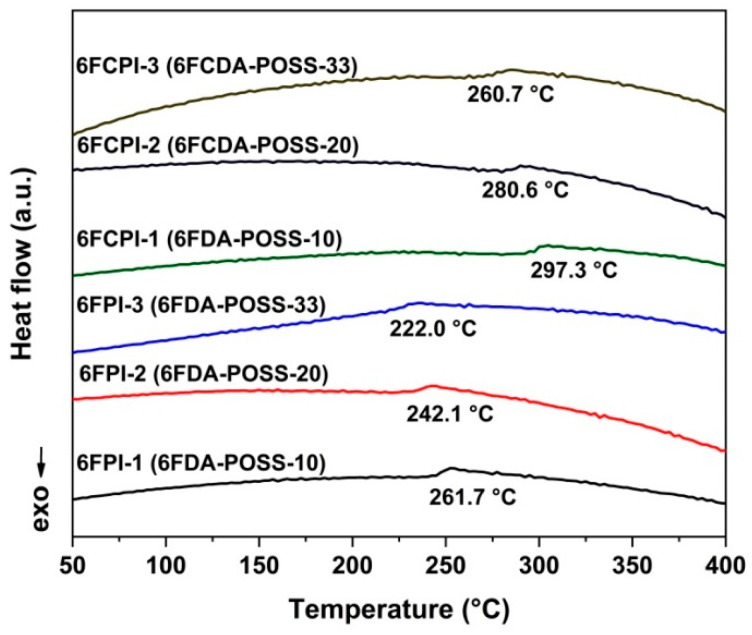
DSC curves of PI films.

**Figure 9:**
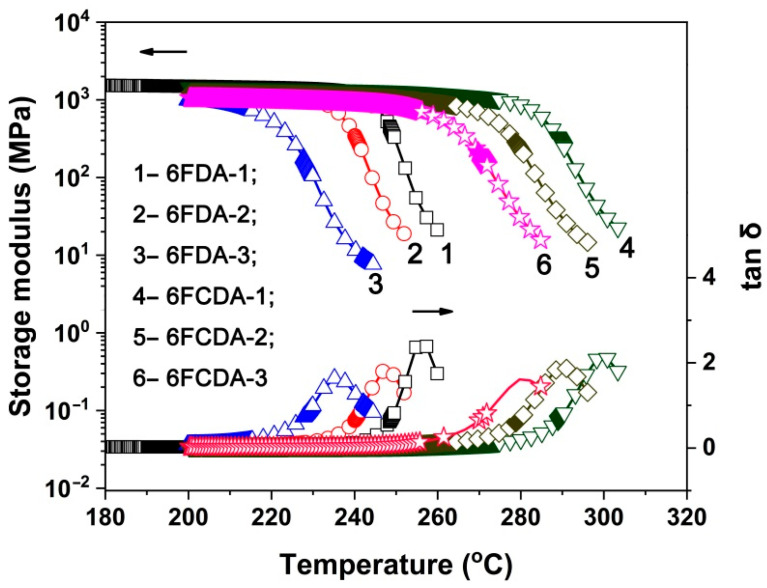
DMA curves of PI films.

Figure 8 and Figure 9 show the DSC and DMA plots of the PI films, respectively, revealing the glass transition temperatures determined using DSC (T_g,DSC_) and DMA (T_g,DMA_), as shown in Table 3. For the two test methods, the obtained T_g_ values showed the following trends: First, with the increasing POSS contents in the PI films, the T_g_ values showed a gradually decreasing trend. For example, the T_g,dsc_ value of PI-ref2 (6FCDA-BDAF) was 311.5 °C, while the values for the 6FCPI-1~6FCPI-3 films with 10–33 mol% of the DABA-POSS component in the diamine units decreased to 297.3 °C, 280.6 °C and 262.7 °C, respectively. A similar trend was observed for the analogous 6FPI films. The decrease in the T_g_ was mainly due to the existence of the relatively bulky POSS side chains in the DABA-POSS diamine, which might act as a plasticizer, making the PI molecular chain segments easy to move at elevated temperatures. Secondly, the 6FCPI films with xanthene ring structures showed higher T_g_ values than the 6FPI films with linear structures in the dianhydride moiety. For example, the T_g,dsc_ value of the 6FCPI-1 film was 297.3 °C, which was 35.6 °C higher than that of the 6FPI-1 (T_g,dsc_ = 261.7 °C). This was mainly due to the fact that the xanthene ring structures endowed the 6FCPI molecular chains with good planar characteristics and relatively high molecular chain packing densities. This efficiently prohibited the free motion of the 6FCPI molecular chain segments at high temperatures.

**Figure 10:**
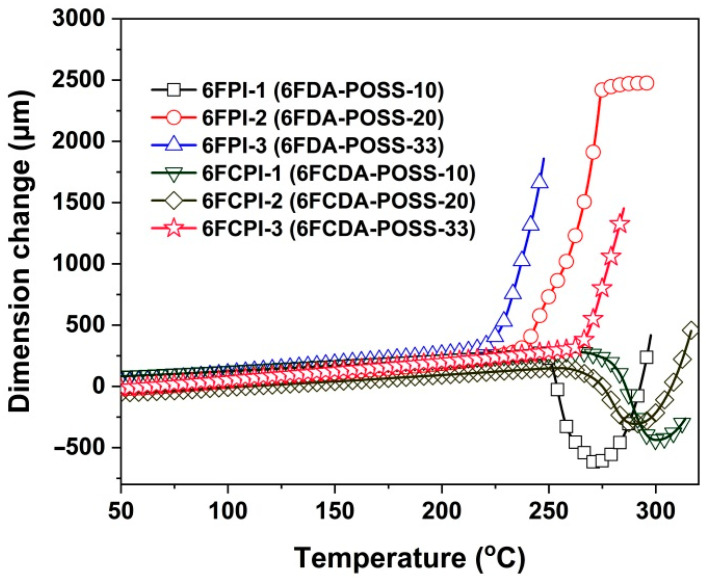
TMA curves of PI films.

Figure 10 shows the TMA plots of the PI films, by which the linear coefficients of the thermal expansion (CTE) values of the polymers were confirmed, as shown in Table 3. It can be seen from the figure that the PI films show different dimensional change behaviors with increasing temperatures. For the PI systems with a relatively high T_g_, such as 6FPI-1 (T_g_ = 261.7 °C), 6FCPI-1 (T_g_ = 297.3 °C) and 6FCPI-2 (T_g_ = 280.6 °C), the films shrank first and then expanded again. However, for the PI systems with a relatively low T_g_, such as 6FPI-2 (T_g_ = 242.1 °C) and 6FPI-3 (T_g_ = 222.0 °C), they only showed thermal expansion behaviors. This was mainly due to the fact that for the PI films with high T_g_ values, since the highest operating temperature (250 °C) during the film-making process did not exceed the T_g_ values, the molecular chains of these polymers were basically in an entangled state. Therefore, when the temperature exceeded the T_g_ of the polymers, the molecular chain segments of the PI films moved and underwent rearrangements, showing a shrinking phenomenon for the films. When the rearrangements of the molecular chains completed, the films showed expansion behaviors with further increases in the temperature. For the PI systems with a low T_g_, because the maximum film-making temperatures have exceeded their T_g_ values, the rearrangements of the molecular chains were completed during the film-making course, so only thermal expansion behaviors were detected in the TMA tests. This phenomenon is very common for the high-T_g_ PI films described in the literature [36].

In addition, it could be deduced from the CTE results of the PI films that with the increasing POSS contents in the PI films, the CTE values of the films showed an increasing trend. For example, PI-ref2 (6FCDA-BDAF) had a CTE value of 50.5 × 10^−6^/K, whereas the CTE values of the 6FCPI-1~6FCPI-3 with 10–33 mol% of DABA-POSS contents in the diamine units increased to 58.6 × 10^−6^/K, 62.2 × 10^−6^/K and 91.9 × 10^−6^/K, respectively. The reason for the increasing CTE values of the copolymer films was consistent with the effects of the structures of the films on the T_g_; that is to say, the bulky POSS side chains acted as a plasticizer at high temperatures, making the PI films prone to thermal expansion at elevated temperatures.

The high-frequency dielectric properties, including the dielectric constant (*D_k_*) and dielectric dissipation factors (*D_f_*) of the PI films at 10 GHz, are shown in Table 3. Basically, all the PI films exhibited low-*D_k_* and low-*D_f_* features, with the *D_k_* values being below 3.0 and the *D_f_* values being around 10 to the power of −3. This feature was mainly ascribed to the combined effects of the highly electronegative hexafluoroisopropylidene units with low molar polarizability and the lateral POSS units with high molar volumes, according to the Clausius–Mossotti equation [37].

### 3.3. Optical Properties

**Figure 11:**
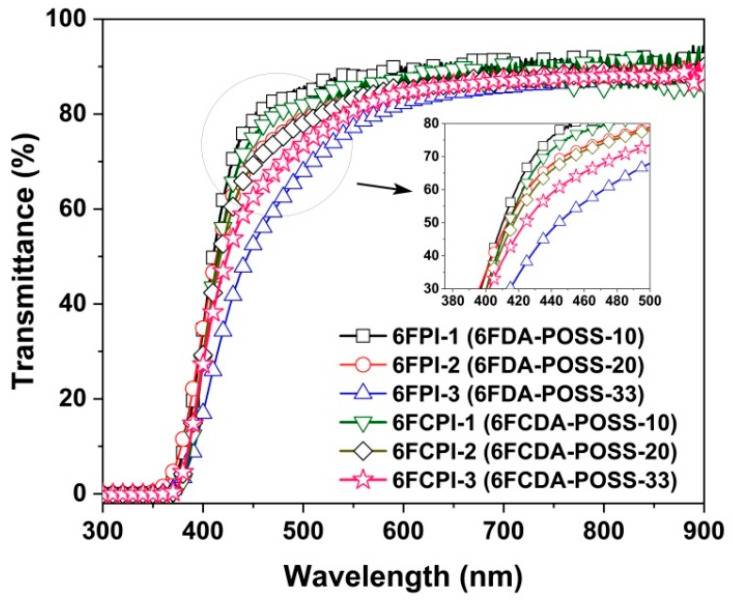
UV-Vis spectra of PI films.

Good optical properties, including high optical transmittance and low yellow indices, are usually essential for the practical applications of PI optical films for spacecraft. Figure 11 depicts the UV-Vis spectra of the PI films, and the optical data are tabulated in Table 4. Overall, the copolymerized PI films showed good optical transparency in the visible light region, with an ultraviolet cut-off wavelength (λ_cut_) below 370 nm, and optical transmittance at a wavelength of 450 nm (T_450_) up to 78.5%. The good optical transparency of the current PI films was mainly due to the coeffects of the highly electronegative hexafluoroisopropylene chains and the bulky POSS side chains with a high molar volume in the PI molecular chains, which effectively inhibited the CT interaction of the PI molecular chains. Compared with the homopolymerized PI reference films, the optical properties of the copolymerized PI films show several typical characteristics. First, the T_450_ values of the PI films were lower than those of the unmodified PI reference films, and the optical transmittance of the films gradually decreased with the increasing POSS contents in the polymers. For example, the T_450_ value of the PI-ref2 film is 84.3%, while the 6FCPI-1~6FCPI-3 films with the incorporation of 10–33 mol% DABA-POSS in the diamine units showed decreasing T_450_ values of 74.9%, 69.3% and 62.6%, respectively. A similar trend was also observed for the 6FPI films. This was mainly due to the fact that DABA-POSS diamine, as a monomer with a high degree of conjugation, enhanced the CT interactions in the PI molecular chains when being incorporated into the molecular structures of the PIs, resulting in the absorption of the visible light.

Secondly, the refractive indices of the PI films were also lower than those of the homopolymerized PI films. For example, the average refractive index (n_av_) value of the PI-ref2 film was 1.5755, while the n_av_ values of the 6FCPI-1~6FCPI-3 films were reduced to 1.5644, 1.5566 and 1.5457, respectively. At the same time, the n_av_ values of the 6FCPI films were higher than those of the analogous 6FPI films. For example, the 6FCPI-3 film showed an n_av_ value of 1.5457, which was higher than that of 6FPI-3 (n_av_ = 1.5372). It is well known that the refractive index of the one polymer is closely related to the molar polarizability (P) and the molar volume (V) of its molecular chains according to the Lorentz–Lorenz equation [38]. The hexafluoroisopropylene group had a low *p* value, while the POSS group had a high V value, thus endowing the PI film with a lower refractive index when they were incorporated into the molecular chain of the PI films. On the other hand, the cyclic xanthene structure in the 6FCDA dianhydride unit endowed the PI molecular chains with good planarity, increased the packing densities of the molecular chains and, thus, reduced the V value, resulting in an increase in the refractive indices of the PI films. For the same reason, the 6FCPI films also showed higher birefringences (Δn) than those of the 6FPI films.

Finally, the CIE Lab color parameters of the PI films were compared. It could be seen that with the increasing DABA-POSS contents in the molecular structures of the copolymerized PI films, the brightness (L*) of the films showed a downward trend, while the yellowness index (b*) and the haze values showed an upward trend. This was the same as the influence factor of the structures of the PI films on the optical transmittance; that is, the incorporation of the highly conjugated DABA-POSS diamine increased the absorption of the visible light by the films. This was different from the synthesis process reported in the literature [31], since in the current work, the aromatic diamine of DABA-POSS containing the POSS unit in the side chain was synthesized first, which was directly used to prepare the copolymerized PIs. In the literature, the PI resin with the reactive –COOH side chain was synthesized first, which was reacted with the amino-terminated POSS compound to afford the final PIs. The process for the PI preparations in the current work was more easily accessible than that reported in the literature [31], but the optical properties of the final PI films might be negatively affected. This was mainly due to the fact that although the DABA-POSS diamine was a white crystal immediately after recrystallization and de-colorization treatment, it was easily oxidized to a brownish-red appearance when exposed to air or light during the storage. Therefore, DABA-POSS usually needed to be stored away from light in a closed container filled with nitrogen. Even so, slight oxidative discoloration was inevitable, which directly affected the optical properties of the final PI films. The process described in the literature [31] avoided the use of the DABA-POSS, and although the synthesis route was complicated, the optical properties of the final PI films were better. Nevertheless, the optical properties of the PI films obtained in this study are still acceptable, and the optical properties of the PI films could be improved by purifying the mass-prepared PI resins several times, which will be investigated in our next work.

### 3.4. AO Erosion Properties

**Figure 12:**
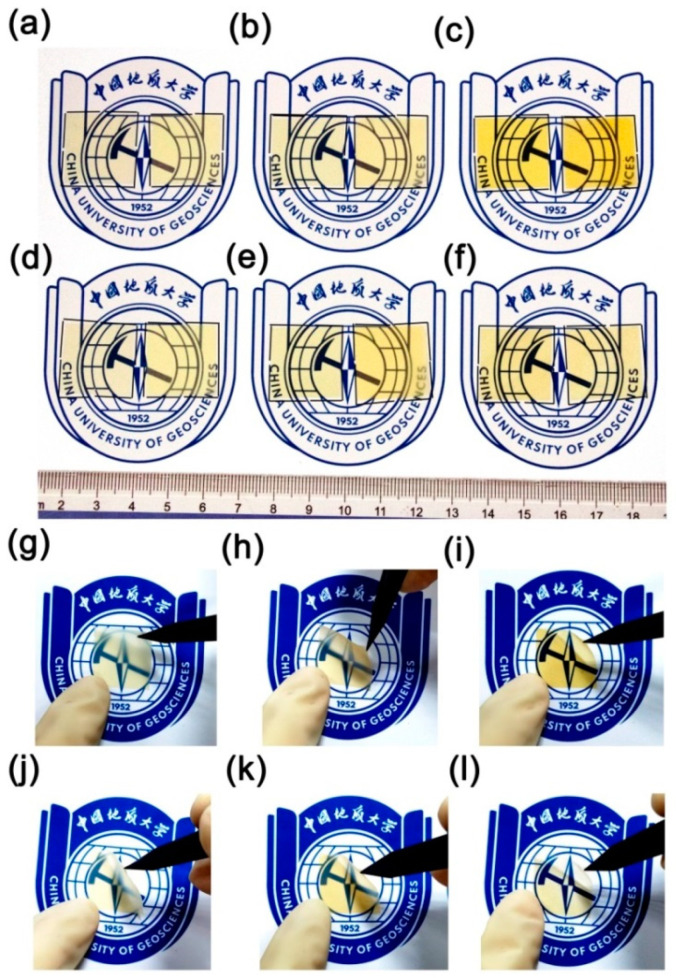
Appearance of PI films before and after AO exposure (doze: 5.0 × 10^20^ atoms/cm^2^). (**a**–**c**): 6FPI-1~6FPI-3 films: left: pristine film; right: film after AO exposure; (**d**–**f**): 6FCPI-1~6FCPI-3 films: left: pristine film; right: film after AO exposure; (**g**) 6FPI-1-AO; (**h**) 6FPI-2-AO; (**i**) 6FPI-3-AO; (**j**) 6FCPI-1-AO; (**k**) 6FCPI-2-AO; (**l**) 6FCPI-3-AO.

The AO erosion properties of the PI films were further studied in the ground simulation facility with the total fluence of 5.0 × 10^20^ atoms/cm^2^. Our previous work has shown that both the PI-ref1 and PI-ref2 films degraded significantly when irradiated with AO [10]. After being irradiated with AO at a dose of 5.0 × 10^20^ atoms/cm^2^, the PI films showed erosion yields (*E_y_*) of 2.88 × 10^−24^ cm^3^/atom for PI-ref1 and 2.77 × 10^−24^ cm^3^/atom for PI-ref2, respectively, which were at the same level as the standard Kapton^®^ films (*E_y_* = 3.0 × 10^−24^ cm^3^/atom). Therefore, PI-ref1 and PI-ref2 films cannot be directly used in AO atmospheres in LEO. It has been well established in the literature that the AO resistant ability of the common PI films could be significantly improved by introducing POSS units into the molecular or composition structures of PI films via the chemical copolymerization or physical blending procedures [39]. The enhancements were based on the fact that the Si elements in the POSS structures could react with AO in situ to form a passivation protective layer. In this study, the effects of AO irradiation on the optical properties of the PI films were first investigated. Figure 12 compares the appearance of the PI films before and after AO irradiation. The PI films after AO exposure were named “6FPI-X-AO” or “6FCPI-X-AO” (X = 1, 2 or 3). It could be seen that for both classes of PI films, AO irradiation caused a decrease in the optical transmittance of the PI films to a certain extent.

**Figure 13:**
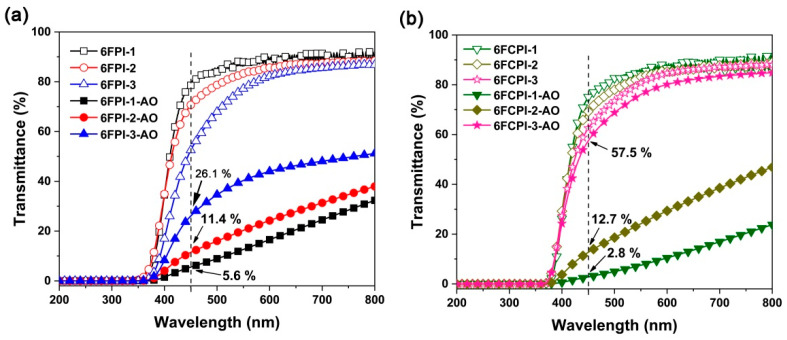
Comparison of UV-Vis spectra of PI films before and after AO exposure. (**a**) 6FPI; (**b**) 6FCPI.

In order to quantitatively investigate the effects of AO irradiation on the optical transmittance of PI films, the UV-Vis spectra of PI films before and after irradiation were tested and compared, and the results are shown in Figure 13. It could be seen that although AO irradiation resulted in the decrease of the transmittance of the PI films, the degree of transmittance decline of the films was significantly different. Surprisingly, for both film systems, the ones with the highest POSS contents (6FPI-3 and 6FCPI-3) showed the lowest T_450_ decline. For example, before and after AO irradiation, the T_450_ values of the 6FPI-3 film decreased from 52.5% to 26.1% for 6FPI-3-AO, which was about 50% of the initial value. For the 6FCPI-3 film, the T_450_ value decreased from 62.6% to 57.5% for the 6FCPI-3-AO film after AO irradiation, and the absolute value decreased by only about 5.1%. To explain this phenomenon, it is necessary to investigate the effects of AO irradiation on the micro-morphologies of PI films.

**Figure 14:**
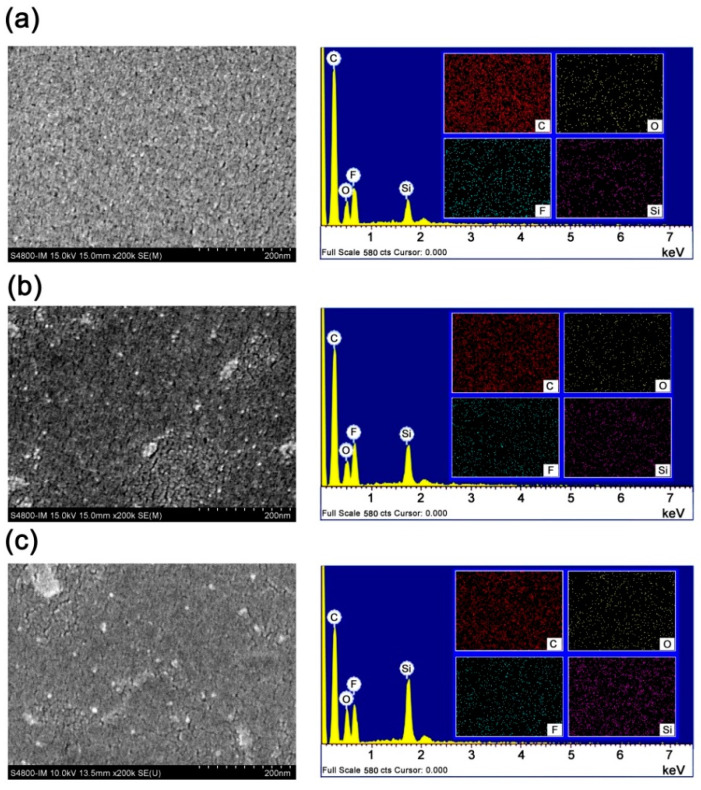
SEM and EDS images of pristine 6FCDA-PI films. (**a**) 6FCPA-1, (**b**) 6FCPI-2 and (**c**) 6FCPI-3.

**Figure 15:**
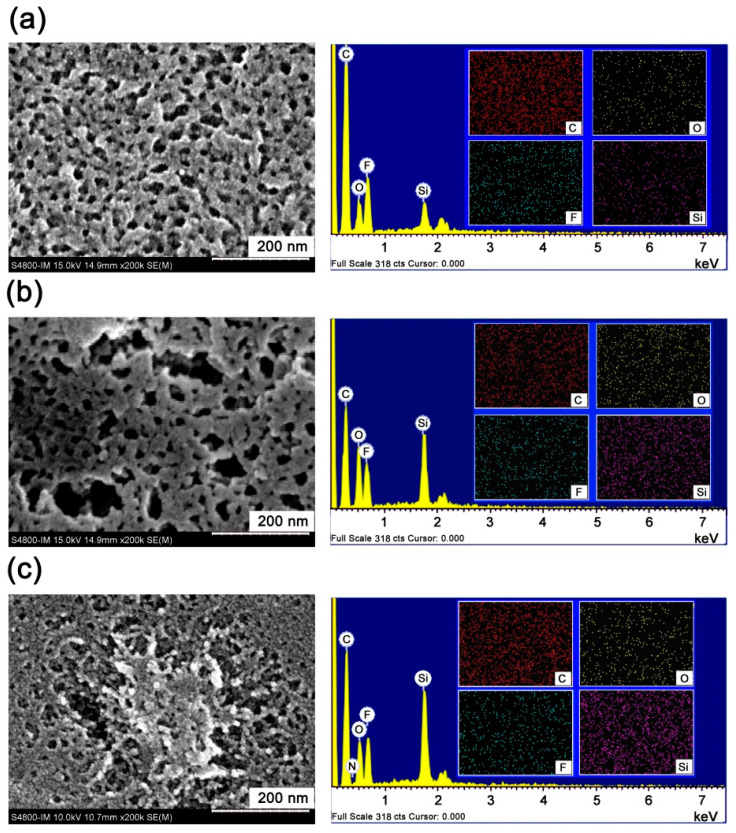
SEM and EDS images of 6FCDA-PI films after AO exposure (5.0 × 10^20^ atoms/cm^2^). (**a**) 6FCPA-1-AO, (**b**) 6FCPI-2-AO and (**c**) 6FCPI-3-AO.

Figure 14 and Figure 15 compared the SEM micro-morphologies of the 6FCPI films before and after AO irradiation, respectively, and the corresponding energy dispersive spectroscopies (EDS) of the PI films are presented. It could be seen that AO irradiation resulted in the rough surfaces of the originally flat PI films. This was especially apparent for the 6FCPI-1 and 6FCPI-2 films with relatively low POSS contents. For the 6FCPI-3 films with the highest POSS contents, AO irradiation also resulted in the same effects for the surface degradation, but the formed layered materials showed significantly better continuity (Figure 15c) rather than the large and discontinuous holes (Figure 15a,b) as for the other two PI films. As for the element compositions of the surface layer, although the EDS tests could only qualitatively show the changes in the element compositions of the PI film surfaces, it could still provide valuable elemental information for the PI films. The ratio of Si and O elements on the surface of the PI films increased significantly, while the ratio of the F and C elements decreased. In summary, the AO irradiation might form the passivation layers of silicon oxide or silicate on the surface of the PI films [40,41]. The passivation layers tended to form partially continuous surfaces when the POSS contents in PI films were high, so that the optical transmittance of the 6FCPI-3 film did not decrease significantly after AO irradiation.

A more accurate elemental composition of the surfaces of the PI films after AO irradiation could be obtained using the XPS tests and the values shown in Table 5. It could be seen that for both PI systems, after AO irradiation, the relative atomic concentrations of the C and F elements on the surface decreased significantly, and the concentration of the N element decreased slightly; however, the concentrations of the O and Si elements increased significantly. For example, for the 6FCPI-3 films with the highest POSS contents, after AO irradiation, the concentration of the C element decreased from 71.97% to 18.07%, the concentration of the O element increased from 15.66% to 52.37%, and the concentration of the Si element increased from 2.33% to 20.20%. It was obvious that in the course of AO irradiation, the Si element in the PI films reacted with the atomic oxygen in situ to form a passivation layer, and the ratio of O to Si in the passivation layer was about 2.40–2.72 for 6FPI and 2.47–2.61 for 6FCPI, respectively. Therefore, it could be inferred that the passivation layer consisted of silicon oxides or silicates.

**Figure 16:**
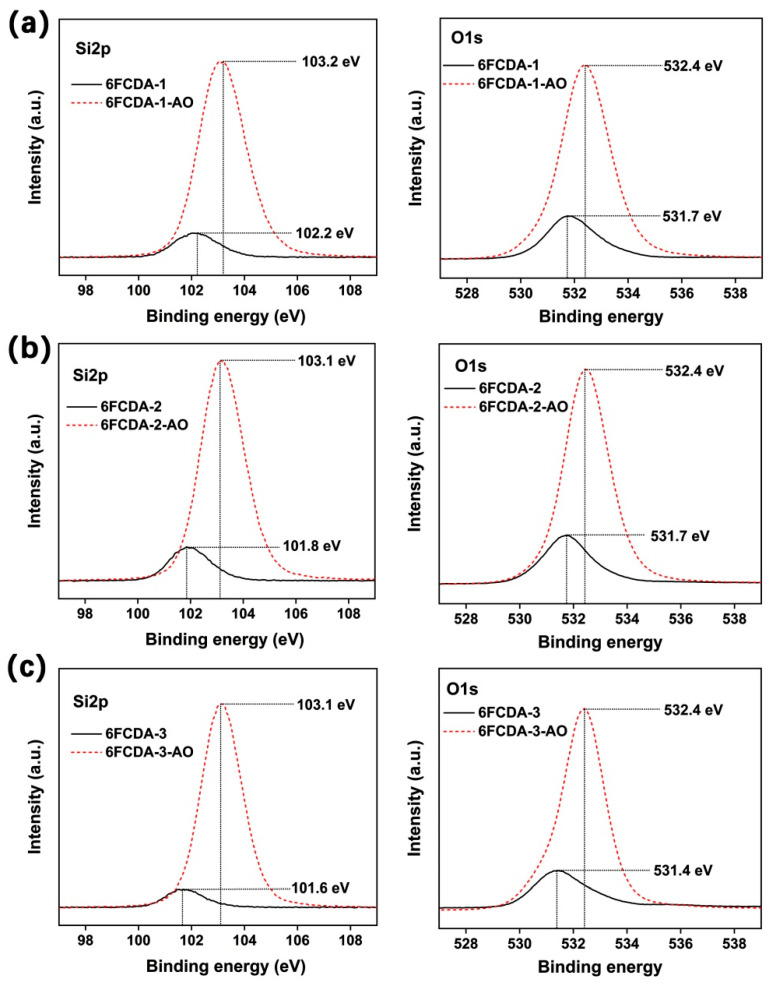
XPS spectra of Si2p and O1s for 6FCPI films. (**a**) 6FCPI-1 and 6FCPI-1-AO; (**b**) 6FCPI-2 and 6FCPI-2-AO; (**c**) 6FCPI-3 and 6FCPI-3-AO.

Figure 16 depicts the changes in the binding energy of the Si2p and O1s for the PI films before and after AO irradiation. As could be seen from the figure that for the three PI films, the binding energies of the Si2p and O1s elements on the film surfaces all increased after AO irradiation. For example, for the 6FCPI-3 film, after AO irradiation, the binding energies of Si2p and O1s on the surfaces of the PI film increased from the initial 101.6 eV and 531.4 eV to 103.1 eV and 532.4 eV, respectively. It has been well established that the increasing shifts in the binding energies of the Si2p and O1s elements usually indicated the formation of higher oxidized species, such as silicon oxides or silicates [2]. The inert passivation layer undoubtedly formed on the surfaces of the currently developed POSS-containing PI films after AO irradiation.

The passivation layers formed on the surfaces of the PI films during the AO irradiation were quite beneficial for protecting the underlying PI films from the further erosion by AO. This also made the weight loss of the current PI films exhibit nonlinear characteristics when they were eroded by atomic oxygen. Under the same conditions, the PI-ref1, PI-ref2 and the standard Kapton^®^ films all exhibited linear weight loss characteristics. The AO erosion rates (*E_y_*) of the 6FPI-3 and 6FCPI-3 films with the highest POSS contents were as low as 0.17 × 10^−24^ cm^3^/atom, which was not only much lower than the standard Kapton^®^ films (*E_y_* = 3.0 × 10^−24^ cm^3^/atom), but also significantly lower than the unmodified reference films, such as PI-ref1 (*E_y_* = 2.88 × 10^−24^ cm^3^/atom) and PI-ref2 (*E_y_* = 2.77 × 10^−24^ cm^3^/atom) (Table 6). This indicated that it was feasible to incorporate the POSS units into the molecular structures of the PI films to improve their AO resistance. In addition, it is worth noticing that the current POSS-linked PI films showed higher *E_y_* values than those reported in ref. [13], which contained POSS compounds as the additives. For example, the 6FPI-3 and 6FCPI-3 films showed an *E_y_* value of 0.17 × 10^−24^ cm^3^/atom, which was much higher than that of the FPI-25 film with 25 wt% of trisilanolphenyl-POSS (TSP–POSS) in the composite films (*E_y_* = 0.031 × 10^−24^ cm^3^/atom). This is mainly due to the different POSS contents in the PI films. For the 6FPI-3 and 6FCPI-3 films, POSS was covalently linked in the structures, while for the FPI-25 nanocomposite films, 25 wt% of the POSS was introduced into the composites. However, it could be anticipated that the nanocomposite films using the current 6FPI-3 and 6FCPI-3 as the matrix and POSS compounds as the filler might possess very low E_y_ values. This will be investigated in our future work. At last, the *E_y_* value for the current 6FPI-3, which was thought to have the similar structure with that of CORIN^®^, was 0.17 × 10^−24^ cm^3^/atom. This value was higher than the value of 2.05 × 10^−26^ cm^3^/atom with an AO fluence of 9.3 × 10^20^ atoms/cm^2^ for the CORIN^®^ material, as reported in the literature [31]. This might be due to the difference in the AO irradiation fluence used in the two studies and might also be related to the difference in the composition structures and physical properties of the prepared PI films.

## 4. Conclusions

As a kind of space material with the best comprehensive properties so far, CORIN^®^ will be more and more widely used in space explorations in the future. In this study, we attempted to replace the 6FDA component in the CORIN^®^ material with 6FCDA, with the aim of improving the T_g_ of the material and reducing its CTE while maintaining its intrinsic thermal resistance, optical properties and AO resistance as much as possible. The test data showed that this target has been achieved using the methodology described. If the 6FPI-3 synthesized in this study was taken as the reference, the thermal resistance and high-temperature dimensional stability of the developed 6FCPI-3 with a similar structure have been significantly improved. Meanwhile, good optical and AO-resistant characteristics were achieved by the polymer. If the CORIN^®^ material reported in the literature was used as the standard, the 6FCPI-2 material developed in this study showed better thermal properties, including a higher T_g_ (280.6 °C versus 266.0 °C for CORIN^®^) and a lower CTE (62.2 × 10^−6^/K versus 68.0 × 10^−6^/K for CORIN^®^). Although the 6FCPI-2 film exhibited inferior optical properties and AO resistance to the CORIN^®^, it might be remedied by the further modification of the films. Future research will focus on how to further improve the optical transparency of 6FCPI films with potential modification methods such as via the repeated purification of soluble 6FCPI resin, the optimization of the film-making conditions and so on. In addition, how to achieve the continuous biaxially stretching preparation of the 6FCPI films is also one of the focuses in the future.

## Figures and Tables

**Table 1:** Recipes for PI and referenced PI resin synthesis.

PI	6FDA (g, mol)	6FCDA (g, mol)	BDAF (g, mol)	DABA-POSS (g, mol)	NMP (g)	Ac_2_O (g)	Py (g)
6FPI-1	22.2120, 0.05	NA ^a^	23.3303, 0.045	5.0436, 0.005	151.8	30.6	19.8
6FPI-2	22.2120, 0.05	NA	20.7380, 0.04	10.0871, 0.01	158.8	30.6	19.8
6FPI-3	22.2120, 0.05	NA	17.3681, 0.0335	16.6437, 0.0165	168.7	30.6	19.8
6FCPI-1	NA	22.9110, 0.05	23.3303, 0.045	5.0436, 0.005	153.9	30.6	19.8
6FCPI-2	NA	22.9110, 0.05	20.7380, 0.04	10.0871, 0.01	161.2	30.6	19.8
6FCPI-3	NA	22.9110, 0.05	17.3681, 0.0335	16.6437, 0.0165	170.8	30.6	19.8
PI-ref1	22.2120, 0.05	NA	25.9225, 0.05	NA	144.4	30.6	19.8
PI-ref2	NA	22.9110, 0.05	25.9225, 0.05	NA	146.5	30.6	19.8

^a^ Not applicable.

**Table 2:** Inherent viscosities, molecular weights and solubility of PI resins.

PI	Molecular Weight ^a^			Solubility ^b^				
	M_n_ (×10^4^ g/mol)	M_w_ (×10^4^ g/mol)	PDI	NMP	DMAc	DMF	DMSO	CPA
6FPI-1	8.47	14.67	1.73	++	++	++	++	+
6FPI-2	5.05	9.51	1.88	++	++	++	++	+
6FPI-3	3.30	6.09	1.84	++	++	++	++	−
6FCPI-1	7.93	14.34	1.81	++	++	++	+	−
6FCPI-2	6.75	12.59	1.86	++	++	++	+	−
6FCPI-3	4.60	9.05	1.97	++	++	++	−	−
PI-ref1	29.64	37.59	1.27	++	++	++	++	++
PI-ref2	9.92	15.30	1.54	++	++	++	+	−

^a^ M_n_: number average molecular mass; M_w_: weight average molecular mass; PDI: polydispersity index, PDI = M_w_/M_n_; ^b^ ++: soluble; +: partially soluble; −: insoluble; DMSO: dimethyl sulfoxide; CPA: cyclopentanone.

**Table 3:** Thermal and dielectric properties of PI films.

Samples	T_g,DSC_^a^ (°C)	T_g,DMA_^a^ (°C)	T_5%_^a^ (°C)	T_max_^a^ (°C)	R_w750_^a^ (%)	CTE ^a^ (×10^−6^/K)	D_k_^c^	D_f_^c^
6FPI-1	261.7	257.4	509.7	560.3	54.3	69.2	2.84	0.0076
6FPI-2	242.1	246.8	483.0	553.0	50.2	90.9	2.71	0.0068
6FPI-3	222.0	235.2	485.3	552.3	46.1	94.5	2.68	0.0068
6FCPI-1	297.3	300.9	477.4	502.5 (555.0 ^b^)	54.2	58.6	2.86	0.0099
6FCPI-2	280.6	288.5	472.5	505.4 (560.5)	51.2	62.2	2.83	0.0085
6FCPI-3	260.7	282.3	463.3	502.2 (560.0)	50.6	91.9	2.80	0.0083
PI-ref1	264.6	273.5	544.5	566.2	55.3	55.4	2.77	0.0071
PI-ref2	311.5	312.4	516.8	569.0	55.4	50.5	2.92	0.0140

^a^ T_g,DSC_: glass transition temperatures according to the DSC measurements; T_g,DMA_: glass transition temperatures according to the DMA measurements (peaks of tan δ plots); T_5%_: temperatures at 5% weight loss; T_max_: temperatures at the rapidest thermal decomposition rate; R_w750_: residual weight ratio at 750 °C in nitrogen; CTE: linear coefficient of thermal expansion in the range of 125–175 °C; ^b^ T_max_ of the second stage of thermal decomposition; ^c^ D_k_, D_f_: dielectric constant and dissipation factor recorded at 10 GHz, respectively.

**Table 4:** Optical properties of PI films.

Samples	λ_cut_^a^ (nm)	T_450_^a^ (%)	n_TE_^a^	n_TM_^a^	n_av_^a^	Δn ^a^	L* ^a^	a* ^a^	b* ^a^	Haze (%)
6FPI-1	348	78.5	1.5578	1.5514	1.5557	0.0064	95.16	−1.76	7.81	1.01
6FPI-2	342	70.7	1.5454	1.5399	1.5436	0.0055	94.66	−1.50	9.47	3.44
6FPI-3	357	52.5	1.5376	1.5364	1.5372	0.0012	92.43	−1.70	19.92	5.34
6FCPI-1	369	74.9	1.5738	1.5453	1.5644	0.0285	94.86	−1.93	8.64	2.05
6FCPI-2	367	69.3	1.5638	1.5420	1.5566	0.0218	94.44	−1.68	11.51	2.86
6FCPI-3	367	62.6	1.5504	1.5364	1.5457	0.0140	93.54	−1.18	13.76	3.98
PI-ref1	350	85.5	1.5701	1.5520	1.5641	0.0181	95.89	−2.29	5.37	0.24
PI-ref2	370	84.3	1.5873	1.5516	1.5755	0.0357	95.71	−2.54	6.00	0.78

^a^ λ_cut_: cutoff wavelength; T_450_: transmittance at the wavelength of 450 nm with a thickness of 25 μm; n_TE_ and n_TM_: in-plane and out-of-plane refractive indices of the PI films, respectively; n_av_: average refractive indices of the PI films; Δn: birefringence, Δn = n_TE-_n_TM_; L*, a* and b*: see Measurements section.

**Table 5:** XPS results for the unexposed and exposed PI films.

Samples	Relative Atomic Concentration (%)									
	Unexposed Samples					AO Exposed Samples				
	C1s	N1s	O1s	F1s	Si2p	C1s	N1s	O1s	F1s	Si2p
6FDA-1	66.38	3.85	12.37	14.7	2.14	14.27	1.38	56.37	3.21	23.53
6FDA-2	63.37	3.39	13.57	16.73	2.93	13.7	1.36	56.67	2.95	23.32
6FDA-3	64.15	4.12	17.84	6.20	7.69	15.68	2.20	55.58	2.79	20.45
6FCDA-1	67.64	3.75	16.00	8.5	3.54	15.03	1.45	55.46	3.76	21.27
6FCDA-2	68.85	2.64	18.26	3.97	4.81	15.96	0.95	56.06	2.60	22.66
6FCDA-3	71.97	2.27	15.66	4.72	2.33	18.07	1.28	52.37	3.73	20.20

**Table 6:** Erosion yields for the PI films.

Samples	*W*_1_ ^a^ (mg)	*W*_2_ ^a^ (mg)	Δ*W* ^a^ (mg)	*E_y_* ^b^ (10^−24^ cm^3^/Atom)
6FPI-1	16.48	15.21	1.27	0.51
6FPI-2	14.40	13.56	0.84	0.34
6FPI-3	22.31	21.89	0.42	0.17
6FCPI-1	14.08	12.57	1.51	0.61
6FCPI-2	14.66	13.56	1.10	0.44
6FCPI-3	11.60	11.18	0.42	0.17
PI-ref1	NA ^c^	NA	NA	2.88
PI-ref2	NA	NA	NA	2.77
Kapton^®^	NA	NA	NA	3.00

^a^ *W*_1_: weight of the PI samples before AO irradiation; *W*_2_: weight of the PI samples after AO irradiation; Δ*W*: weight loss of the PI samples during AO irradiation, Δ*W* = *W*_1_ − *W*_2_; ^b^ erosion yield; ^c^ not applicable.

## Data Availability

Data are contained within the article.
